# ISOGO: Functional annotation of protein-coding splice variants

**DOI:** 10.1038/s41598-020-57974-z

**Published:** 2020-01-23

**Authors:** Juan A Ferrer-Bonsoms, Ignacio Cassol, Pablo Fernández-Acín, Carlos Castilla, Fernando Carazo, Angel Rubio

**Affiliations:** 1 Department of Biomedical Engineering and Sciences, Tecnun-Universidad de Navarra, Manuel de Lardizábal 15, 20018 San Sebastián, Spain; 2 Department of Bioengineering, Facultad de Ingeniería, Universidad Austral, Mariano Acosta, 1611 Buenos Aires Argentina

**Keywords:** Computational biology and bioinformatics, Data integration, Gene ontology, Machine learning, Protein function predictions, Software

## Abstract

The advent of RNA-seq technologies has switched the paradigm of genetic analysis from a genome to a transcriptome-based perspective. Alternative splicing generates functional diversity in genes, but the precise functions of many individual isoforms are yet to be elucidated. *Gene Ontology* was developed to annotate gene products according to their biological processes, molecular functions and cellular components. Despite a single gene may have several gene products, most annotations are not isoform-specific and do not distinguish the functions of the different proteins originated from a single gene. Several approaches have tried to automatically annotate ontologies at the isoform level, but this has shown to be a daunting task. We have developed ISOGO (ISOform + GO function imputation), a novel algorithm to predict the function of coding isoforms based on their protein domains and their correlation of expression along 11,373 cancer patients. Combining these two sources of information outperforms previous approaches: it provides an area under precision-recall curve (AUPRC) five times larger than previous attempts and the median AUROC of assigned functions to genes is 0.82. We tested ISOGO predictions on some genes with isoform-specific functions (*BRCA1*, *MADD*,*VAMP7* and *ITSN1*) and they were coherent with the literature. Besides, we examined whether the main isoform of each gene -as predicted by APPRIS- was the most likely to have the annotated gene functions and it occurs in 99.4% of the genes. We also evaluated the predictions for isoform-specific functions provided by the CAFA3 challenge and results were also convincing. To make these results available to the scientific community, we have deployed a web application to consult ISOGO predictions (https://biotecnun.unav.es/app/isogo). Initial data, website link, isoform-specific GO function predictions and R code is available at https://gitlab.com/icassol/isogo.

## Introduction

Alternative splicing (AS) is a genetic process by which a single pre-mRNA can originate different mature mRNAs (called isoforms or transcripts) by including or excluding exons and introns^1–4^. It is estimated that genes have on average 7 transcripts, that the whole transcriptome there are more than 100,000 AS events^5,6^ and that over 90% of human genes contain one or more isoforms^7–10^.

From a functional point of view, AS is an intriguing process. Some studies show that a large number of sporadic splicing events produce alternative isoforms lowly expressed, and thus may be non-functional noise in the transcription process^11–13^. On the other hand, other studies show and experimentally validate that different isoforms originated by alternative splicing may have distinct or even opposite functions^14,15^. It is known that AS can cause cellular abnormalities that lead to diverse genetic diseases. All the hallmarks of cancer have their counterpart in AS^16–18^. For example, *BRCA1* is a tumor suppressor gene related to breast cancer susceptibility. Its isoform originated from skipping exon 11 (that includes a RAD51 interaction domain) is associated with lacking its ability to repair DNA^19^. AS has also been documented as a factor of the chemoresistance in hematological cancers^20–22^. These examples illustrate that the study of isoform-specific functions is essential to better understand cancer.

In past years, multiple algorithms have predicted gene functions based on functional ontologies, such as the Gene Ontology database (GO)^23^ by using different machine learning techniques^24–29^. These methods are focused on the gene function predictions^30^ and do not distinguish between different gene products for a single gene.

Recently, some promising attempts have been developed to predict biological functions at the isoform-level. These approaches are mainly based on the protein structure (3D model^31,32^ or domains^33^), amino acid sequence and expression^4,29–31^ to associate GO functions to each isoform. Surprisingly, none of the previous algorithms combined RNA expression with structural information. In this work, we combine isoform expression with protein domains to predict the probability of an isoform to perform a given GO function. New methods to study RNA-seq data measure isoform expression much more reliably and can be combined with protein domain information (which is annotated at the isoform level).

In this work, we discovered that the combination of both sources of information -protein domains and expression correlation- increases five-fold the precision of the predictions for genes. We compared the performance of the model with the methodology proposed by Panwar *et al*.^34^, Li *et al*.^5^ and Eksi *et al*.^35^ because they were similar works to ours from an algorithmic point of view. ISOGO was tested on some paradigmatic cases (*BRCA1*, *MADD*, *VAMP7* and *ITSN1*) and on some GO *terms* that are annotated at the isoform level taken from the CAFA3 challenge^36^. In addition, we found that the main isoforms -predicted by APPRIS^37^- were the ones with largest probability of having the function of the gene in an overwhelming percentage.

The final contribution of this proposal is the ISOGO web application (https://biotecnun.unav.es/app/isogo) which provides a convenient framework to consult the probability of an isoform to perform a GO *term*.

## Results

We have developed ISOGO, an inference model that predicts GO functions of coding genes or isoforms by integrating both expression data and protein domains. We restricted our predictions to coding regions precisely to use their protein domains as a source of information.

The underlying reasoning of ISOGO is the following. On the one hand, genes with similar functions show a higher correlation in expression than genes with dissimilar functions^38^. ISOGO tests this fact by comparing the correlation of the expression of an isoform (or a gene) with genes that have or do not have a particular function using a Wilcoxon test (*Correlation method*). On the other hand, protein domains are known to be related to GO functions and, in fact, Interpro provides a relationship of its domains (or Pfam) with GO functions^39^. In ISOGO, we used a regularized logistic regression to predict GO functions based on the protein domains annotated to a coding transcript (*Domain-based regression*). Both predictions are combined (using another logistic regression) to infer the function of a specific gene or isoform (*Combination method*). This way, an isoform is likely to perform a GO *term* if its expression is correlated with the expression of genes annotated to this GO *term*. If the protein coded by the isoform includes some specific domains, this prediction will be reinforced.

**Figure 1:**
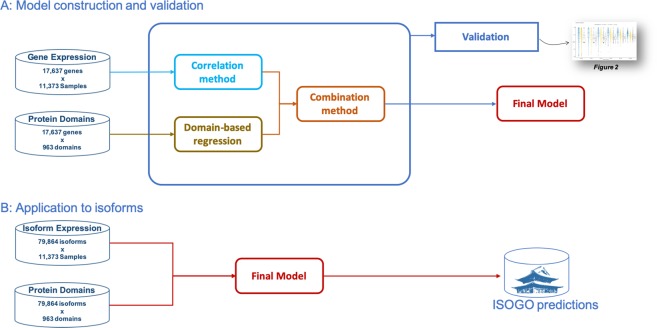
Overall proposal. Train and validation are performed with a train and a test set of genes respectively and the complete prediction model is built with the complete set of genes and finally it is applied to isoforms data achieving the final ISOGO matrix with [79,864 isoforms × 5,777 GO terms].

These predictions must be validated to state their performance. We have implemented a standard training set/test set procedure to quantify the precision and sensitivity of the predictions. Figure 1 shows a graphical representation of the proposal. Firstly, the model using gene information is built. This model was validated on gene functions. Then, this model is applied to predict isoform functions. The final output is the ISOGO matrix with 5,777 *GO terms* predictions for 79,864 coding isoforms. Figures S1–S4 of *Supplementary documentation* contains a detailed graphical representation of the procedure to validate and generate the model.

Isoform expression was collected from^40^ where Kallisto was applied to samples of The Cancer Genome Atlas (TCGA) resulting in 79,864 transcripts and 19,637 genes using 11,373 TCGA samples from 33 different cancer types. We also tested the algorithm using expression profiles from 200 normal samples from 32 different tissues of 122 donors^41–43^ and from the CCLE database (923 cell-lines corresponding to 24 cancer types). CCLE expression was also collected from^40^. Protein domains were obtained from the Pfam protein families database^44^. Data are available through the repositories cited in the *Methods* section.

### Performance of GO predictions for genes

We evaluated ISOGO performance at the gene level by means of the Precision-Recall (PR) and the Receiver Operating Characteristic (ROC) curves. Specifically, we calculated the median of the area under the receiver operator curve (AUROC), the median of the area under the precision recall curve (AUPRC) and the number of functions with perfect performance i.e. AUROC and AUPRC equal to one (Table 1). We compared these results with Panwar *et al*.^34^ that performed a similar approach, where 2,129 *GO terms* were predicted with a median AUROC of 0.641 and a median AUPRC of 0.011. These 2,129 GO functions were annotated to a minimum of 20 and a maximum of 300 genes. We found an improvement both in terms of AUROC and AUPRC values (Table 1).

**Table 1:** Overall performance of each method and Panwar *et al*.^34^ AUROC column shows the median of the AUROC; AUPRC displays the median of the AUPRC for each method; #perfect column indicates the number of total functions with perfect performance. AUROC* and AUPRC* columns show the median of the AUROC and the AUPRC for those GO terms annotated to more than 20 and less than 300 genes to make a fair comparison with Panwar *et al*.

Method	AUROC	AUPRC	#perfect	AUROC*	AUPRC*
Correlation method	0.739	0.0087	11	0.733	0.0122
Domain-based regression	0.657	0.0293	147	0.673	0.0473
Combination method	0.816	0.0414	191	0.815	0.0646
Panwar *et al*.^34^				0.641	0.011

Our results -excluding from this comparison GO *terms* annotated to less than 20 genes not included in^34^- for the *Correlation method* outperform this method in terms of AUROC (0.733 vs 0.641) whereas in AUPRC both methods have similar performance (0.012 vs 0.011). Li *et al*.^5^ also uses expression to build the model and its AUROC is similar both to ISOGO and Panwar ones (mean AUROC of 0.67, Figs. S5 and S6).

For the *Combination method*, both the AUROC (0.816 vs 0.641) and, especially, the AUPRC (0.0646 vs 0.011) outpace Panwar’s results. The integration of both sources of information is the most important reason why ISOGO provides better predictions than previous approaches.

C*orrelation method* outperforms *Domain-based regression* in terms of AUROC. On the contrary, *Domain-based regression* betters C*orrelation method* if AUPRC is considered. Domain-based regression is better to avoid false positives, i.e. it can be very precise for low values of recall. Correlation method provides better precision than Domain-based regression for large values of recall (Fig. S7). In addition, Domain-based regression is able to perfectly predict 147 *GO terms*. For these genes, the presence of some domains is sufficient to state univocally their functions. Correlation method achieves this perfect performance only for 11 genes. The *Combination method* outperforms them in all the three aspects (AUROC value of 0.816, AUPRC value of 0.0414 and 191 *GO terms* with perfect performance).

**Figure 2:**
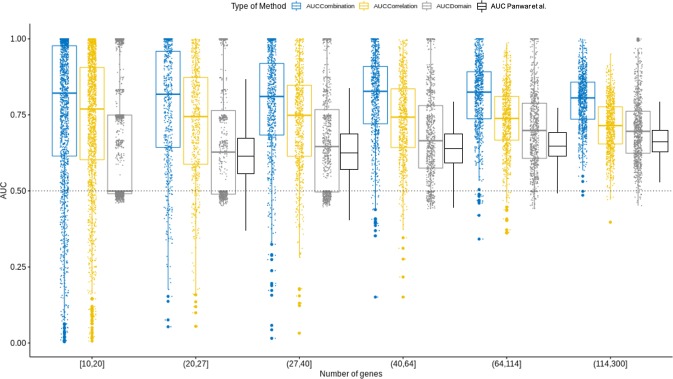
AUROC comparison, depending on the number of genes per GO term. Blue boxplots correspond to the Combination method, yellow ones to the Correlation method and grey ones to the Domain-based regression. Black boxplots correspond to the result in Panwar *et al*. A dotted black line is included to show the baseline for a random classifier (AUROC = 0.5).

**Figure 3:**
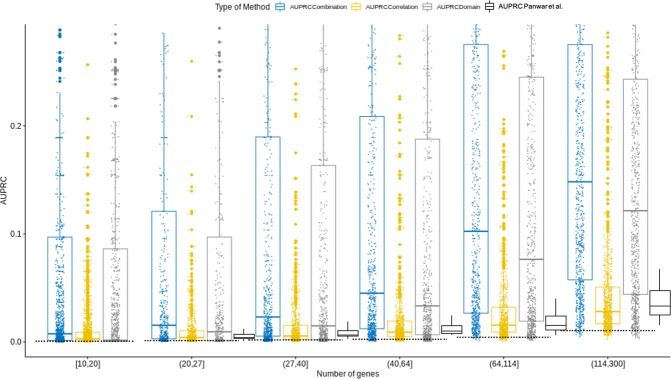
AUPRC comparison, depending on the number of genes per GO term. Legend as for Fig. 2 (blue boxplots are combination method, yellow ones are Correlation method, grey ones are Domain-based regression and black ones are the result from Panwar *et al*.). The dotted black line represents the AUPRC of a random classifier. This value depends on the number of genes per category.

Colored boxplots in Figs. 2 and 3 show a comparison based on AUROC and AUPRC of the three methods, grouped by number of annotated genes per category – [10, 20], (20, 27], (27, 40], (40, 64], (64, 114] and (114, 300] –. The number of genes for each bin is taken from^34^ to ease the comparison with this reference. In both Figs. 2 and 3, black boxplots show the result of Panwar *et al*.^34^ proposal. The *Combination method* outperforms both the *Correlation* and the *Domain-based regression* methods for any GO category size. The *Correlation method* also outperforms *Domain-based regression* for any category size in terms of AUROC.

We compared the results of applying ISOGO to the three GO ontologies: cellular component (CC, 509 functions), molecular function (MF, 884 functions) and biological process (BP, 4384 functions). Table 2 shows the median AUROC, the median AUPRC and the percentage of functions predicted with perfect performance for each ontology. For all ontologies, the performance of the methods follows the pattern displayed in Table 1: the *Correlation method* is better in terms of AUROC than the *Domain-based regression* but worse in terms of AUPRC and number of functions with perfect annotations, except for *Molecular Function* where *Domain-based regression* has better AUROC than *Correlation method*. The *Combination method* outperforms either the *Correlation* and the *Domain-based regression* methods in AUROC, AUPRC and percentage of functions with perfect annotations (Figs. S8 and S9). Molecular function predictions are better than biological processes.

**Table 2:** Overall performance of each method grouped by function ontologies. AUROC columns show the median of the AUROC. AUPRC columns display the median of the AUPRC for each method. %perfect column indicate the percentage of functions predicted with perfect performance for each ontology.

Method	Cellular Component			Molecular Function			Biological Process		
	AUROC	AUPRC	%perfect	AUROC	AUPRC	%perfect	AUROC	AUPRC	%perfect
Correlation method	0.785	0.0132	0.39%	0.727	0.0065	0.22%	0.737	0.0087	0.15%
Domain-based regression	0.685	0.0536	2.94%	0.749	0.1929	7.91%	0.640	0.0216	1.41%
Combination method	0.864	0.0958	4.91%	0.881	0.1890	9.62%	0.801	0.0308	1.85%

Some of the domains in the Pfam database have unknown functions (“Domains of Unknown Functions” named DUFxx where xx is a number). We tested if these domains were used to predict GO *terms*. In this case, the domain will probably have a function related with the predicted GO term. In Table S1 we have included these domains with their Pfam and Interpro description and the top 5 GO *terms* with the most reliable predictions for which they were used as predictors variables.

### Performance of GO predictions for isoforms

Validating isoform predictions is a challenge as a ground-truth dataset of isoform functional annotations is not yet available. Even in the few cases where GO *terms* are annotated to specific proteins, there is still doubt on whether the experiment focused specifically on the annotated protein or rather on the family of proteins coded by a gene. Nevertheless, we propose two approaches to validate the results at an isoform-level: (i) comparing GO predictions with genes with known isoforms specific functions and (ii) using other transcript-level information (APPRIS and CAFA3) as indirect validation sources.

### GO predictions for genes with known isoform specific functions

We will illustrate the performance of ISOGO using some examples from the literature (genes *BRCA1*, *MADD*, *VAMP7 and ITSN1*) with known isoform-specific activities.

**Figure 4:**
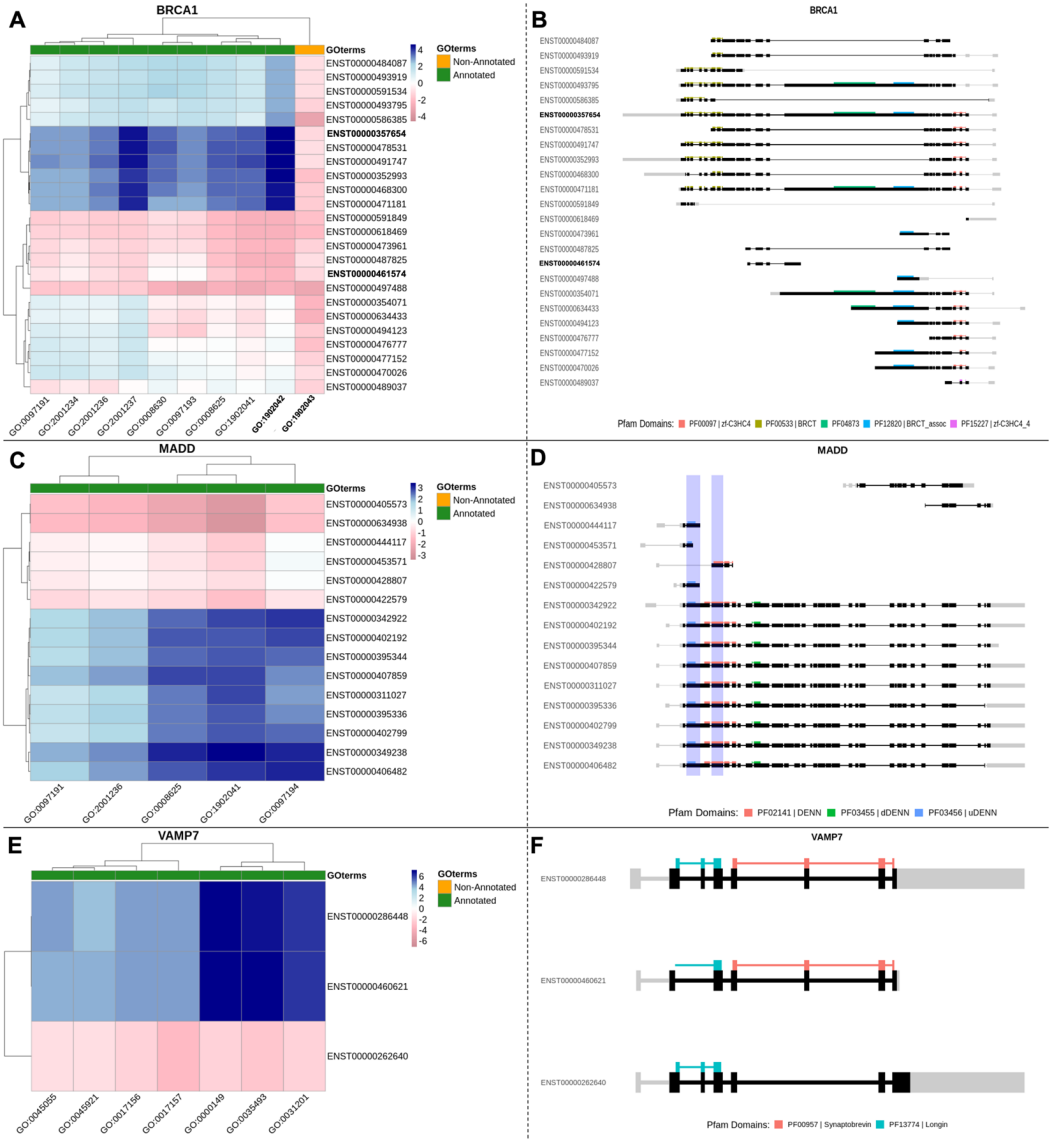
Panels (A,C,E) show heatmaps of the difference between the ISOGO and the expected logits of an isoform having a function, where larger values are represented in blue and smaller values in red. The x-axis of each heatmap picture displays the corresponding studied functions for each gene (Table S2). The functions are related to apoptosis in the case of BRCA1 and MADD -panels (A,C)- and related to exocytosis and SNARE machinery in the case of VAMP7 (panel E). Annotated and non-annotated functions are marked in green and orange respectively on the top of each heatmap. Panels (B,D,F) show the isoform structure and position of protein domains for BRCA1, MADD and VAMP7 respectively. Coding regions are marked in black while 5′ UTR and 3′UTR are colored in grey. Panels (C,D) show that isoforms that include both exons 13 and 16 -shaded blue- have larger logit for the GO functions. Panels (E,F) show that alternative splicing in VAMP7 changes the functions of SNARE machinery and exocytosis.

To state whether a gene or isoform is assigned to a GO *term* or not, we compared the ISOGO and the *expected logits* of having the category. We define the *expected logit* of having a *GO term* as the log of the number of genes that are annotated to the corresponding *GO term* divided by the number of genes that do not have the function. In the following figures (Fig. 4A,C,E), predicted logits that are larger and smaller than expected are shown blue and red, respectively.

In the Introduction, we mentioned the paradigmatic case of *BRCA1*, a tumor suppressor gene whose alternative splicing is related to functional changes. *BRCA1* is annotated, among others, to GO:1902042 (“*negative regulation of extrinsic apoptotic signaling pathway via death domain receptors*”). Isoform ENST00000357654 indeed has this function^19^ and its predicted logit is −2.42 whilst the expected logit is −6.71. On the contrary, ENST00000461574 does not have this function and the corresponding ISOGO logit is −9.10 (with the same expected logit). We tested the predictions of either of the isoforms for the opposite function GO:1902043 (“*positive regulation of extrinsic apoptotic signaling pathway via death domain receptors*”) whose expected logit is −7.17. The predicted logits are −8.87 and −9.10. Therefore, ISOGO correctly predicts the GO:1902042 for the first isoform (as known by literature) to be more likely and the low likelihood of its opposite function is also coherent. Interestingly, neither of these *GO terms* were predicted as likely for the second isoform. Figure 4A shows a heatmap of the difference between the ISOGO and the expected logits for the splice variants of *BRCA1* in apoptosis-related functions. In this image, larger values are represented in blue and smaller values in red. Figure 4B shows the structure and position of protein domains for all the splice variants of *BRCA1*. In this image, it can be shown that isoform ENST00000461574, is much shorter than ENST00000357654 and does not include many of its protein domains. Figure 4A includes several GO functions related to apoptosis. The description of these functions, as well as the ones displayed in the other panels, are included in Table S2.

The MAP Kinase Activating Death Domain (MADD) gene also changes its function owing to AS. According to^45^, the presence of both exons 13 and 16 is positively related to apoptosis. These exons have been shaded in Fig. 4D. Figure 4C shows a heatmap of the difference between the ISOGO and the expected logits for different GO functions (Table S2). The estimated logits are significantly larger in MADD isoforms that include both exons 13 48– exons 2 t and 16 (all the isoforms from row seventh onwards).

Vesicle Associated Membrane Protein 7 (VAMP7) regulates SNARE machinery and exocytosis^46^. In the full-length VAMP7 isoform –ENST000002864o 4 encodes the Longin domain whereas the exons 5 to 8 encodes the Synaptobrevin domain, the domain implicated in the SNARE machinery^44^. Skipping of exon 6 –ENST00000262640– breaks the Synaptobrevin domain. This isoform is not expected to perform functions related to exocytosis and SNARE machinery (Table S2). Figure 4E,F show that this is indeed the case: GO predictions for ENST00000262640 show that this isoform is not related to exocytosis or SNARE complex.

Finally, we tested the ISOGO predictions of two isoforms of gene *ITSN1* that are known to perform opposite functions^47,48^. ISOGO predictions are concordant with the results from the literature. This analysis is described in the supplementary material (Fig. S12).

### Indirect transcriptome-wide validation: APPRIS and CAFA3

**Figure 5:**
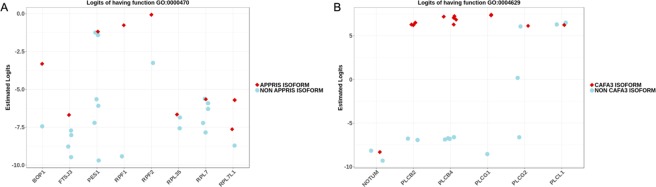
(**A**) Estimated logits for APPRIS and non-APPRIS isoforms. The y-axis displays the logits for all the isoforms of genes annotated to GO:0004629 (maturation of LSU-rRNA). APPRIS Transcripts are shown as red diamonds and other transcripts as blue circles. (**B**) Estimated logits of CAFA3 isoforms. In this case, the y-axis displays the logits for the genes annotated to GO:0004629 (phospholipase C activity). CAFA3 annotations as red diamonds and other transcripts as blue circles.

Not all the coding isoforms of a gene are equally important. Some of them, are tissue-specific or appear only in disease conditions and others can be considered as “transcription noise”^13^. APPRIS is an algorithm to predict which is the most representative isoform for a gene^37,49^. Assuming that the APPRIS annotation is correct, it is sensible to accept that the APPRIS isoform should be the most likely to perform the functions annotated to its corresponding gene (Fig. S10). This is indeed the case: the isoforms with the highest logit for functions annotated to the gene are the APPRIS isoforms in 5745 out of 5777 functions. Figure 5A shows an illustrative example: the x-axis includes the genes annotated to GO:0000470 (“*maturation of LSU-rRNA*”). The expected logit of this function is −7.58. For each gene, the y-axis represents the predicted logits for each of their isoforms. The APPRIS major isoforms have the largest logits for this function, and for most of the annotated functions.

On the other hand, the CAFA3 challenge^36^ provides proteins associated with GO *terms*. Despite the assignment to a specific protein, it is difficult to ensure if the experimental method to make this inference was truly isoform-specific. Nevertheless, it is reasonable to assume that the CAFA3 protein-GO *term* assignations will usually have larger logits than assignations to other protein products of the same gene. By using the *Biomart* R package^50^ we related the proteins included in the CAFA3 challenge with isoforms. It is important to point out that these data were not used in training: none annotation used to train the ISOGO model is isoform-specific. We run a procedure similar to the one in APPRIS to test whether the highest logits of functions assigned to genes are the CAFA3 assignations. A total of 4,135 functions were tested and we found that 3,765 fulfilled this hypothesis (Fig. S11). Figure 5B shows GO:0004629 (“*phospholipase C activity”)* (expected logit equal to −6.48) that follows this pattern.

### ISOGO app

**Figure 6:**
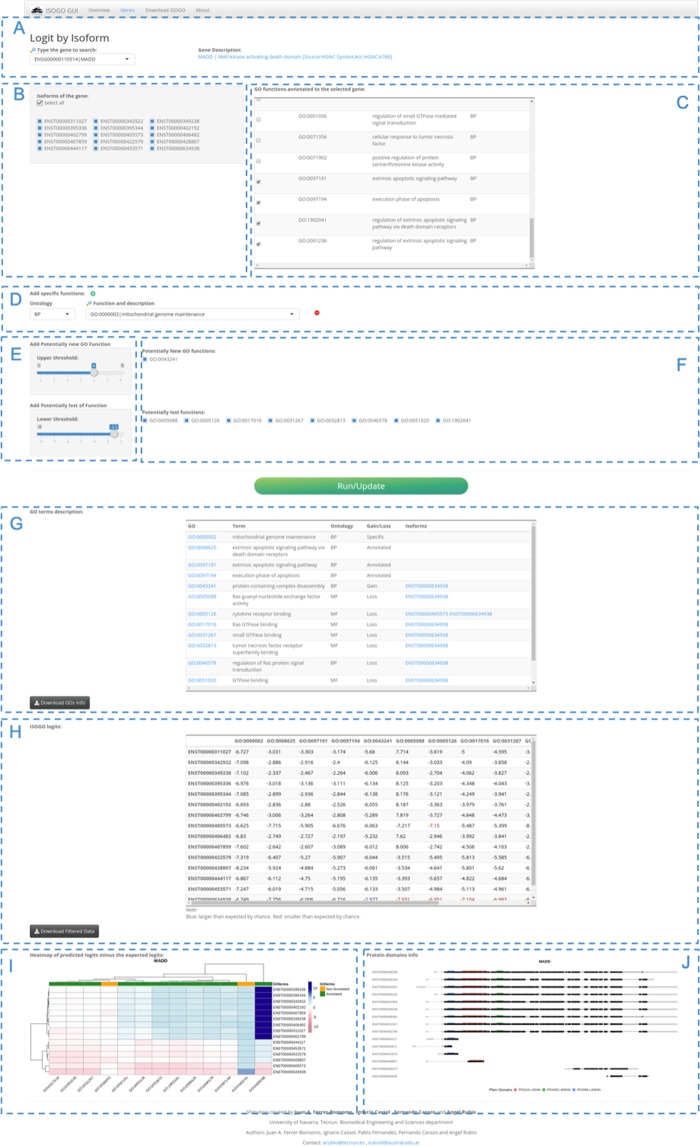
Screenshot of ISOGO web application main page. (**A**) Gene input and a brief description of it. (**B**) Checkbox list of the isoforms of the selected gene. (**C**) List of the genes annotated to the selected gene. (**D**) Option to add manually any GO term to the analysis. (**E**) Upper and lower thresholds set up. (**F**) hide/show list of filtered GO terms. (**G**) GO terms description and its isoforms gained and loss. (**H**) ISOGO table. (**I**) heatmap of the difference between the ISOGO values and the expected logits (**J**) Splice variants structure and protein domains position.

We have developed a web application to share the result with the scientific community (https://biotecnun.unav.es/app/isogo). Figure 6 shows a screenshot of the main window. Given a gene selected by the user (panel A), the main panel of the app returns a table with the description of potential gained or loss *GO terms* (panel G), a second table with the ISOGO logits of the isoforms of the gene having the previous GO *terms* (panel H), a heatmap of the difference between the corresponding ISOGO logit and the expected logits (panel I) and an image of the structure of the isoforms and the position of their protein domains (panel J). The *GO term* description table also shows whether a specific GO *term* is a gained or lost, and which isoforms win/lose it. Both output tables can be downloaded from the app.

The app considers an annotated GO term as a lost function for a given isoform if the difference between the ISOGO and the expected logit is smaller than the lower threshold. Conversely, a non-annotated GO term is considered to be a gain of function if the difference between the ISOGO and the expected logits is larger than the upper threshold. Users can modify both thresholds (panel E). Moreover, users can hide from the output tables and figures any of these GO *terms* (panel F).

Besides, users can add to the output tables and figures any GO *term* annotated to the gene (panel C) or any GO *term* (panel D). In the same way, users can remove from the output tables and figures any isoform annotated to the gene (panel B).

## Discussion

We have developed ISOGO, a set of methods implemented in a web application to predict GO functions at isoform level. It combines structural information (domains) with expression data. The integration of both sources of information improves the overall performance if compared with using each source of information independently.

ISOGO exploits recent algorithms to estimate isoform concentrations. This may be one of the reasons why the expression method alone outperforms previous attempts to predict isoform functions. On the other hand, the *Wilcoxon test* used to predict the function is fast and efficient. We also tried the KS test –the workhorse of GSEA enrichment analysis^51^– but its performance was worse (AUROC: 0.61. Data not shown).

In spite of having a median AUROC of 0.82, or even 0.88 for molecular functions, the predictions are not precise: median AUPRC is 0.0414. The reason is that the number of genes annotated to a given *GO term* is far smaller than the number of non-annotated ones, i.e. the categories are unbalanced. As a consequence, the AUPRC is small and increases for more populated categories (Fig. 3). Nevertheless, our proposal outperformed previous studies^5,34^ providing an AUPRC that is more than five times larger. Furthermore, by applying the *Combination method*, we have perfect predictions on 191 *GO terms*.

Interestingly, even if a gene or isoform is incorrectly predicted to have a function, these false positives are still valuable on their own: these isoforms are coexpressed with genes that have their predicted functions and/or have similar domains. Therefore, these false positives are likely to have a close relationship with their predicted functions. Continuing with the example of BRCA1, GO:0031441 (“*Negative regulation of mRNA 3*′*-end processing”*) has a large logit. We found that, in spite of not being annotated, several references relate *BRCA1* to this GO *term*^52–54^.

It could be argued that, since some of the used annotations of GO terms were electronically-inferred, ISOGO returns what was already predicted by other algorithms. To test this hypothesis, we run ISOGO using only experimental annotations and found that, for all methods (*Correlation*, *Domains* and *Combination*), AUROCs values increased ~2%. In other words, since ISOGO has better AUROC with no electronically-inferred data, ISOGO is not predicting what was already known. Moreover, using electronically-inferred functions decreases the unbalance of the classes and, therefore, results in terms of AUPRC improve.

Pawar *et al*. and Eksi *et al*. use a “multiple instance learning” based approach: each gene is considered as a “bag of isoforms” and at least one of them must perform the annotated functions. This approach requires an iterative process to make the predictions. We used a simpler approach: build the prediction model with gene expression and protein domains and extend it to isoforms. In Eksi *et al*.^35^, the differences in the iteration of the multiple instance learning approach only affect the third decimal (AUROC turns from 0.728 to 0.730) for the best formulation of the instance learning. In fact, only 2 out the 5 possible formulations outperform the standard approach and the AUROC increases 0.002 in the best case.

We selected TCGA expression data to estimate the correlations across the different genes. We tested if using other data sources for expression (such as CCLE or normal tissues) change the predictions. In both cases (CCLE and normal tissues) the AUROC and AUPRC were similar to the ones in TCGA. For CCLE the median AUROC and the median AUPRC were 0.706 and 0.0072 respectively. For normal tissues these values were 0.725 and 0.0082. The results are closer comparing TCGA and normal tissues than comparing TCGA and CCLE. It seems that the effect of being a cell-line is more important than the relationship of the dataset with cancer. Results are shown in Tables S3 and S4.

GO functions have strong dependences: if a gene or isoform is annotated to a GO *term*, it is also annotated to all the ancestors of the function in the ontology. As a consequence, the probability of a gene (or isoform) to have a certain function must be smaller or equal than the probabilities of its ancestors. Predictions that do not follow this rule are termed as “inconsistent”^55^ or that have a lack of “consensus” among GO *terms*^56^ that must be “reconciled”^57^. Our model predicts each GO *term* independently and, therefore, there can appear inconsistencies. We fixed it by using a custom algorithm, termed *Coherence technique* (additional material). This method only slightly improved the AUROC because most predictions were already consistent and, therefore, the algorithm did not change them. As the *Coherence technique* requires ten times more computation time and average AUPRC values drop from 0.16 to 0.12 we did not include the *Coherence technique* in our final model.

The main problem to perform isoform function prediction is the lack of ground truth. We have used indirect methods to state the quality of the predictions, namely, check the performance with genes and literature validation of specific isoforms that are well-annotated. Also, we focused on the APPRIS isoforms and found out that APPRIS logits were larger than other isoforms as expected. Additionally, using a dataset with functions annotated to specific isoforms (CAFA3 challenge), we also got convincing results.

The final contribution is a probability matrix with 5,777 GO *terms* prediction for 79,864 coding isoforms. This data can be easily consulted in the ISOGO web application, which will be helpful for researchers in the complex task of deciphering isoform-specific functions.

## Methods

### Data sources

ISOGO was built and validated using the following data. RNA-Seq expression was collected from^40^ where Kallisto was applied to samples of The Cancer Genome Atlas (TCGA) using the GENCODE24 as reference transcriptome. Isoform expression was measured in TPMs. The expression of a gene was calculated by summing up the expression of all its isoforms and no expression filters were applied. After filtering out non-coding isoforms, the expression data is a matrix of 79,864 transcripts (corresponding to 19,637 genes) from 11,373 TCGA samples.

*Gene-GO terms* associations were downloaded from the Ensembl genome database project (data version v84: March 2016)^58^. We included only GO functions annotated to a minimum of 10 and a maximum of 299 genes resulting in 5,777 GO *terms*.

Protein domains from the Pfam protein families database^44^ were also downloaded from Ensembl (v84). We used domains that appear in at least 6 and no more than 500 genes (total number of domains: 963). Using the *Biomart* R package^50^ we built the isoform-protein domains annotations. To train the model, we considered that each gene has all the protein domains included in some of its isoforms.

### Prediction methods

For all the prediction methods (correlation, domain-based and combination), we selected 17,637 genes for training and the remaining 2,000 genes to evaluate their performance (test set). To extend these results to isoforms, each model was rebuilt using the complete set of genes.

### Correlation method

It has long been stated the relationship between semantic similarity in the GO annotation and correlation of gene expression^38^, i.e. genes with similar functions tend to be coexpressed. As a consequence, two given genes with high correlation across different conditions will likely share the same or similar *GO terms*.

In order to use the coexpression of genes as a proxy to predict GO functions, we computed the *Spearman correlation* coefficient of each gene pair resulting in a matrix of correlations *P*_g,g_ (size genes × genes). Pairs of genes annotated to the same function tend to have larger correlation than pairs of genes non-annotated to the same function. In order to test if a gene “i” have a particular function “j”, we computed a *Wilcoxon test* with a corresponding row of the *P*_gg_ comparing the genes annotated to the “j” *GO term* with genes non-annotated to it -excluding the gene itself. Previous algorithm returns a matrix with the Wilcoxon z-scores whose size is genes × functions (17,637 × 5,777 for the train set, 2,000 × 5,777 for the test set and 19,637 × 5,777 for the complete set). To apply the previous result to isoforms, we computed the Spearman correlation for each isoform-gene pair resulting in a matrix (size 79,864 × 19,637). The *Wilcoxon test* is applied to the rows of this matrix as done with genes resulting in a 79,864 × 5,777 matrix of z-scores. The whole procedure is explained in Figs. S1 to S4 of the supplementary material.

We developed a vectorized function that takes advantage of the sparse nature of the Gene GO associations. This implementation is several orders of magnitude faster than the standard R implementation. The code for this function is available in the Gitlab page of Isogo.

### Domain-based regression

Protein domains give important clues on the specific function of a protein. Ensembl provides isoform-specific annotation of the protein domains using different methodologies (Pfam^44^, Interpro, Panther, etc.). We focused on the Pfam domains and applied an *elastic-net regularized logistic linear model* that predicts for each gene or isoform the logit of having a GO function. The *glmnet* R package^59^ was used to perform this task, applying 10 fold cross-validation. We run 5,777 different logistic regressions, one for each GO function. The regularization λ parameter was selected to provide the best cross-validation AUROC. As we wanted to predict functions by the presence of the domains (not by their absence) we imposed the coefficients of the regression to be non-negative.

To evaluate its performance, we applied the previous model to the test set of genes (not used in the training stage (Figs. S1 and S2). After applying cross-validation, we built a model with the complete set of genes and then applied it to isoforms achieving the estimated logits of each isoform having a specific function (Figs. S3 and S4).

### Combination method

Correlation and protein domain methods use independent information sources to predict GO *terms*. Both results can be joined to get “the best of both worlds”. We integrated them by applying a *Bayesian logistic regression*^60^. We have selected this technique instead of a standard logistic regression to avoid convergence problems with perfectly separable classes. In this logistic regression, the design matrix includes an intercept term, the z-scores of the correlation method, the logits of the domain method, their products and their square values (to account for second-order interactions). The output is the predicted logit of having a function. As in the previous cases, we built a prediction model using the training set and then applied to the test set to estimate its performance with new data (Figs. S1 and S2). The final model is built using the complete set of genes and applied to isoforms, obtaining the final matrix that holds the estimated logit of each isoform having a particular function (Figs. S3 and S4).

### Runtime evaluation

ISOGO was performed on a PC HP Z240 Tower Workstation, Intel Xeon CPU E3-1270 3.80 GHz. RAM 32 Gb using Windows 10 operating system. The overall computing time to build the models using the gene train dataset was 6:53:23 hs. (12:29 mins for *Correlation method*, 5:58:53 hs for *Domain-based regression* and 42:01 mins for *Combination method*). The 86% of the overall calculation elapsed in *Domain-based regression* to build 5,777 logistic regressions by running *glmnet*.

## Materials

### Data and code

Available on https://gitlab.com/icassol/isogo.

## Supplementary information

Supporting Information.
