# Supplementary material for: ISOGO: Functional annotation of protein-coding splice variants
Source: Sci Rep. 2020 Jan 23;10:1069. doi: 10.1038/s41598-020-57974-z (PMC6978412; doi:10.1038/s41598-020-57974-z)
Supplement: Supplementary file 1 — Supporting Information. [file 41598_2020_57974_MOESM1_ESM.pdf]

Supplementary Information

ISOGO: Functional annotation of protein-coding splice variants

Juan A. Ferrer-Bonsoms[1,†], Ignacio Cassol[2,†], Pablo Fernández Acín[1], Carlos Castilla[1] Fernando Carazo[1] and Ángel Rubio[1,*]

[1]Department of Biomedical Engineering and Sciences, Tecnun, Manuel de Lardizábal 15, 20018 San Sebastián, Spain. [2]Department of Bioengineering, Facultad de Ingeniería, Universidad Austral, Mariano Acosta 1611, Buenos Aires, Argentina. * To whom correspondence should be addressed.

† These authors contributed equally to this work.

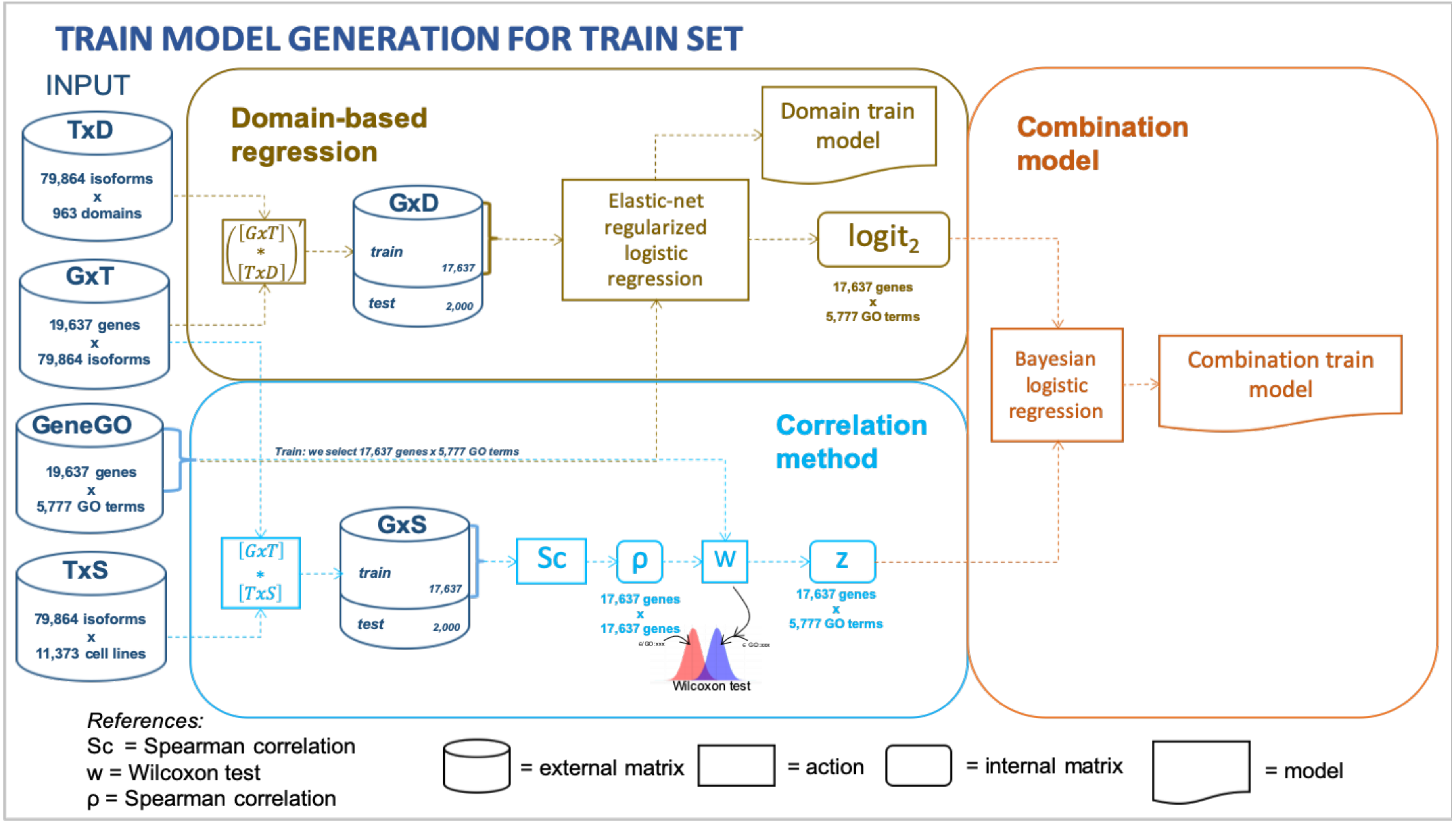

Figure S1: ***Input****: the isoforms-domains annotation (TxD), the isoforms-genes annotation (GxT), the genes-function annotation (GenesGO) and the expression at isoform level of TCGA cell lines (TxS).* ***Domain-based regression:*** *From TxD and GxT we get the genes-domain annotation (GxD). Then an elastic-net regularized logistic regression model is built using the train set achieving the $logit_2$ matrix and the domain train model.* ***Correlation method****: gene expression is calculated from TxS and GxT. We compute a spearman correlation for each gene pair of the train set getting the matrix P. Then, in order to test if a gene "i" has a function "j" we applied a Wilcoxon test with the corresponding row of the matrix P comparing genes annotated to function "j" with the remaining genes achieving the z-scores matrix.* ***Combination method****: Previous scores –$Logits_2$ and z-scores matrices– are combined by a logistic regression achieving the final combination train model for train set.*

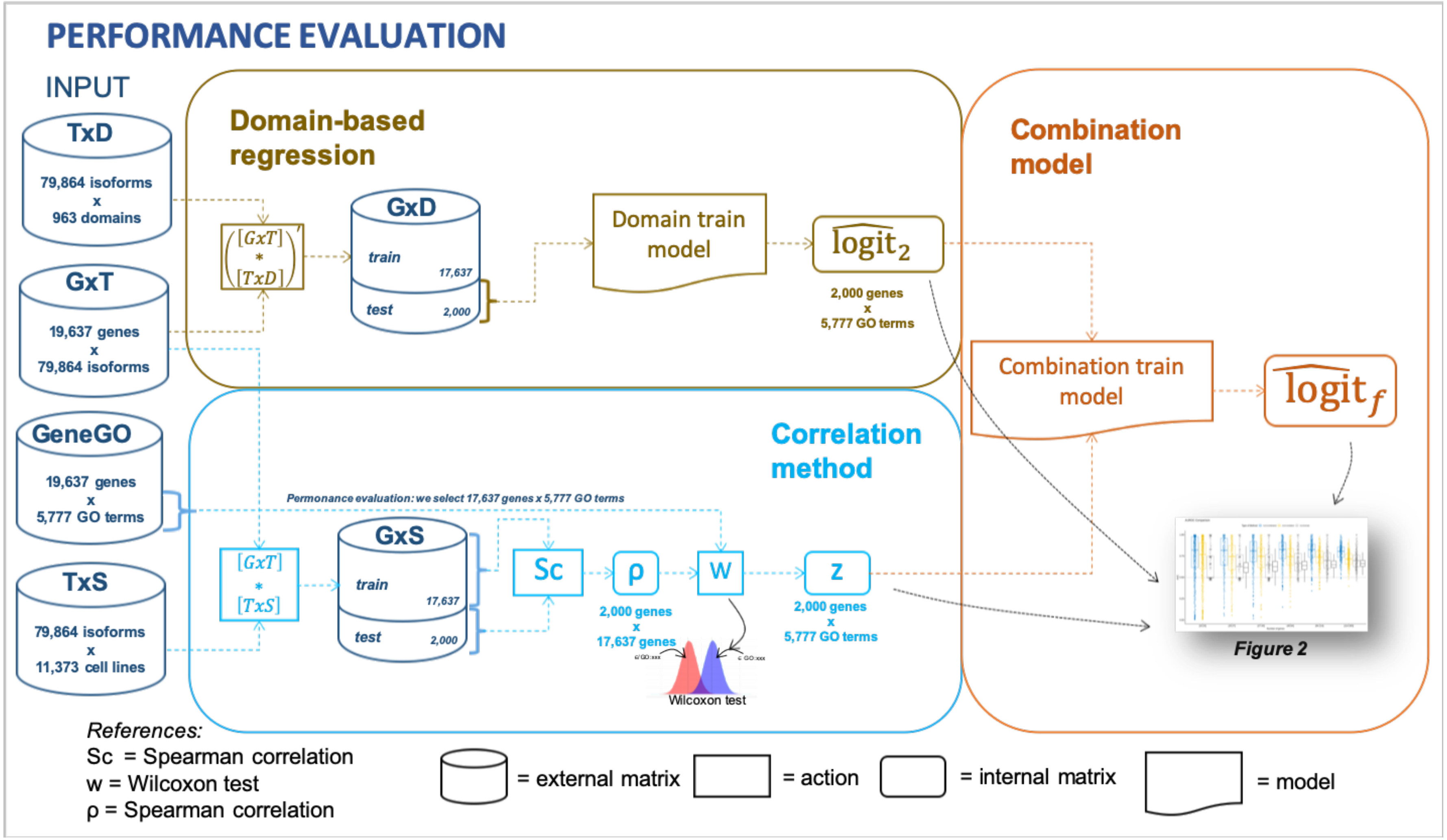

*Figure S2: **Input**: the isoforms-domains annotation (TxD), the isoforms-genes annotation (GxT), the genes-function annotation (GenesGO) and the expression at isoform level of TCGA cell lines (TxS). **Domain-based regression:** From TxD and GxT we get the genes-domain annotation (GxD). Then we applied the Domain train model to the test set achieving the matrix $\widehat{logit}_2$. **Correlation method**: gene expression is calculated from TxS and GxT. Then, we calculate the spearman correlation between each pair of the test and train set achieving the matrix P (size 2,000 x 17,637). In order to test if a gene "I" of the test set has a function "j" we applied a Wilcoxon test with the corresponding row of the matrix P comparing genes annotated to function "j" with the remaining genes achieving the z-scores matrix (size 2,000 x 5,777). **Combination method**: We applied the combination train model for train set to previous scores $-\widehat{logit}_2$and Z matrices obtaining the final matrix $\widehat{logit}_f$.*

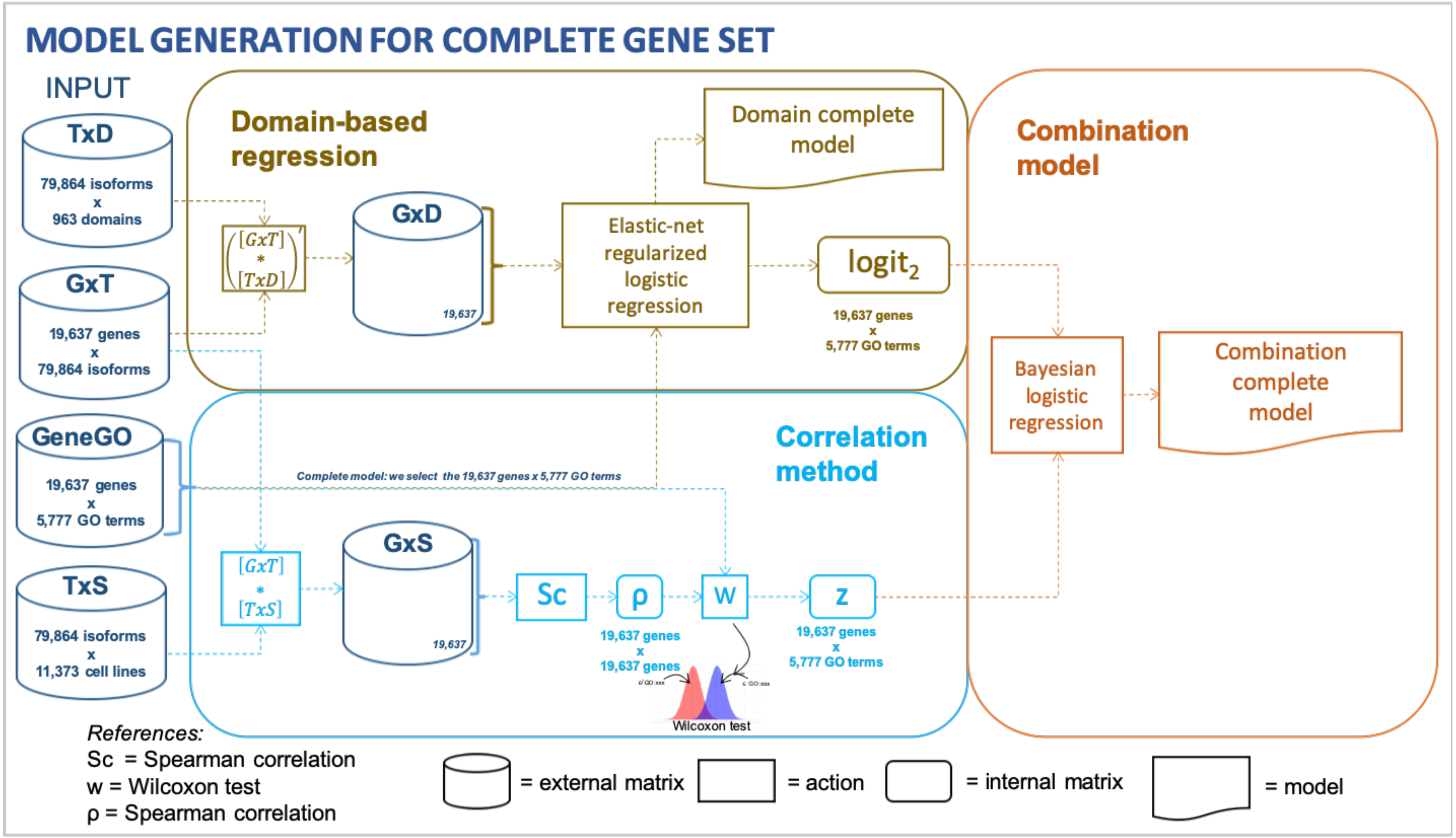

Figure S3: ***Input****: the isoforms-domains annotation (TxD), the isoforms-genes annotation (GxT), the genes-function annotation (GenesGO) and the expression at isoform level of TCGA cell lines (TxS).* ***Domain-based regression:*** *From TxD and GxT we get the genes-domain annotation (GxD). Then an elastic-net regularized logistic regression model is built using the complete set achieving the* $logit_2$ *matrix and the domain complete model.* ***Correlation method****: gene expression is calculated from TxS and GxT. Then, we computed the Spearman correlation coefficient of each gene pair resulting on a matrix of correlations P (size 19,637 x 19,637). In order to test if a gene "i" have a particular function "j", we computed a* Wilcoxon test *with corresponding row of the P comparing the genes annotated to the "j"* GO term *with genes non-annotated to it -excluding the gene itself- achieving the z-scores matrix (size 2,000 x 5,777).* ***Combination method****: Previous scores –*$Logits_2$ *and z-socres matrices– are combined by a logistic regression achieving the final combination complete model.*

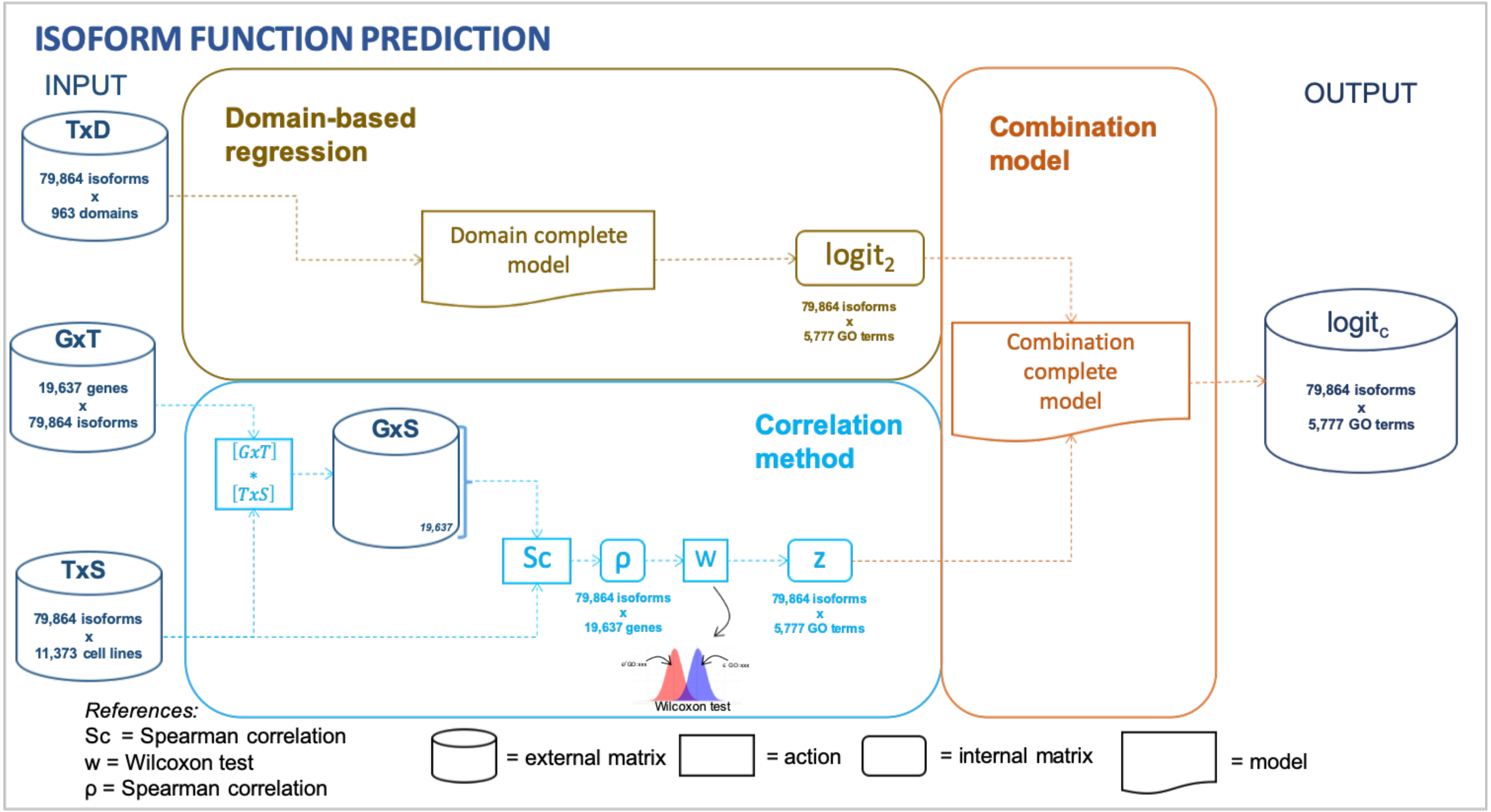

Figure S4: ***Input****: the isoforms-domains annotation (TxD), the isoforms-genes annotation (GxT), and the expression at isoform level of TCGA cell lines (TxS).* ***Domain-based regression:*** *We applied the Domain complete model to isoform-domains annotation achieving the matrix* $\widehat{logit}_2$. ***Correlation method****: gene expression is calculated from TxS and GxT. Then, we computed the Spearman correlation coefficient of each isoform-gene pair resulting on a matrix of correlations P (size 79,864 x 19,637). In order to test if a isoform "i" have a particular function "j", we computed* a Wilcoxon test *with corresponding row of the P comparing the genes annotated to the "j"* GO term *with genes non-annotated to it achieving the z-scores matrix (size 79,864 x 5,777).* ***Combination method****: We applied the combination complete model to previous scores –*$\widehat{logit}_2$*and Z matrices– obtaining the final matrix* $\widehat{logit}_c$ *–The ISOGO matrix–.*

PERFORMANCE OF GO PREDICTIONS FOR GENES

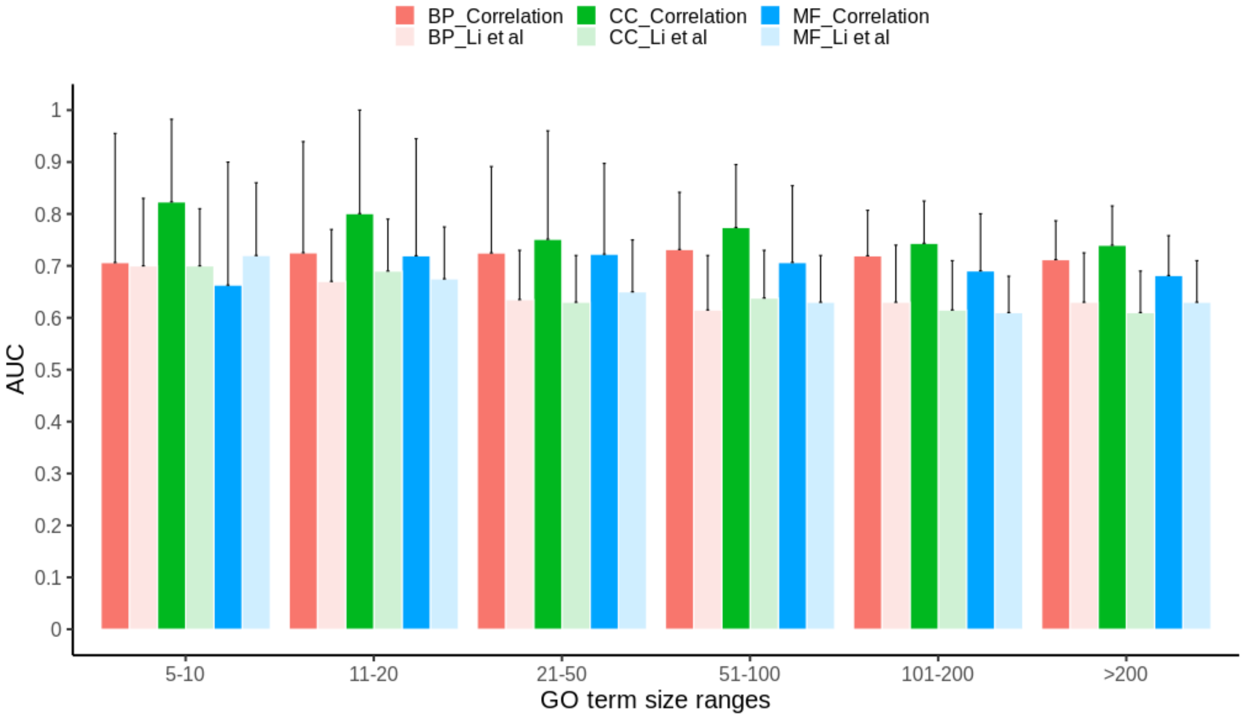

Figure S5: Comparison of the average AUROC obtained in the Correlation method and in the methodology proposed by Li et al. [1]. *The picture shows the average AUROC depending on the number of GO terms annotated and the ontology. We indicate each Gene Ontology branch with red (Biological process), green (Cellular component) and blue (Molecular function). The Li et al. results are indicated in those bars with lighter color. The number of genes for each bin is taken from* [1] *to ease the comparison of both methods.*

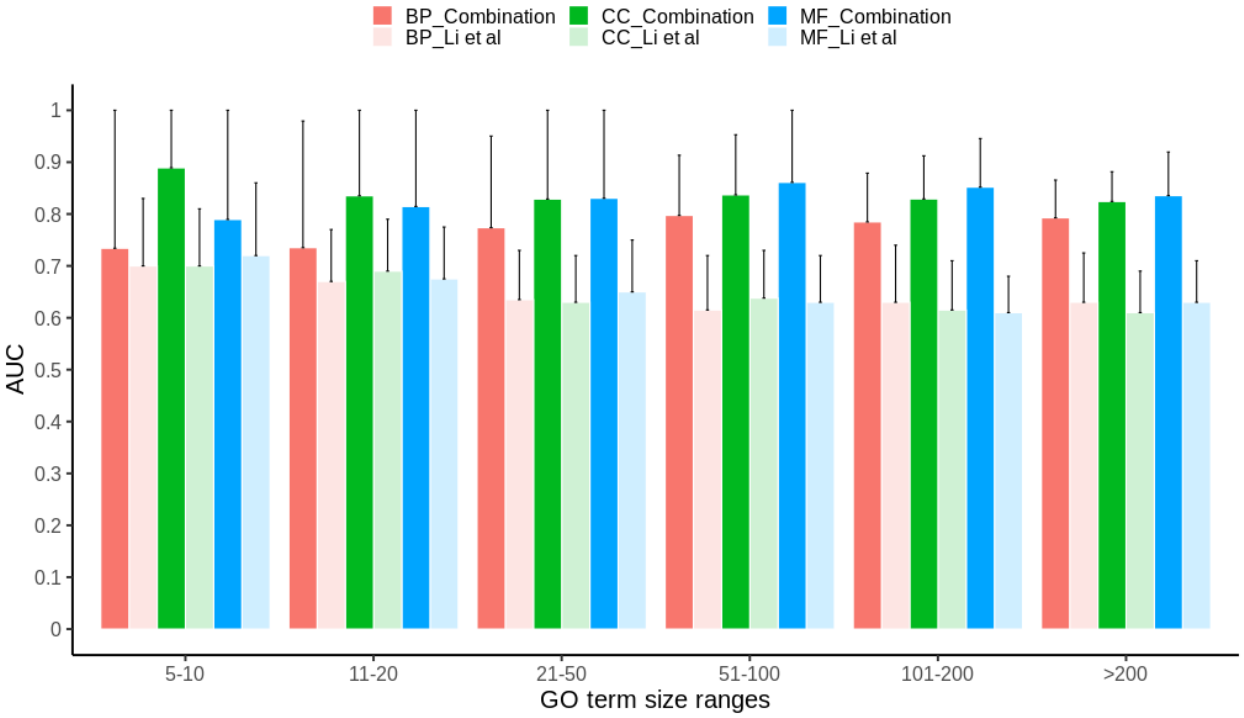

Figure S6: Comparison of the average AUROC obtained in the Combination method and in the methodology proposed by Li et al. [1]. *The picture shows the average AUROC depending on the number of GO terms annotated and the ontology. We indicate each Gene Ontology branch with red (Biological process), green (Cellular component) and blue (Molecular function). The Li et al. results are indicated in those bars with lighter color. The number of genes for each bin is taken from* [1] *to ease the comparison of both methods.*

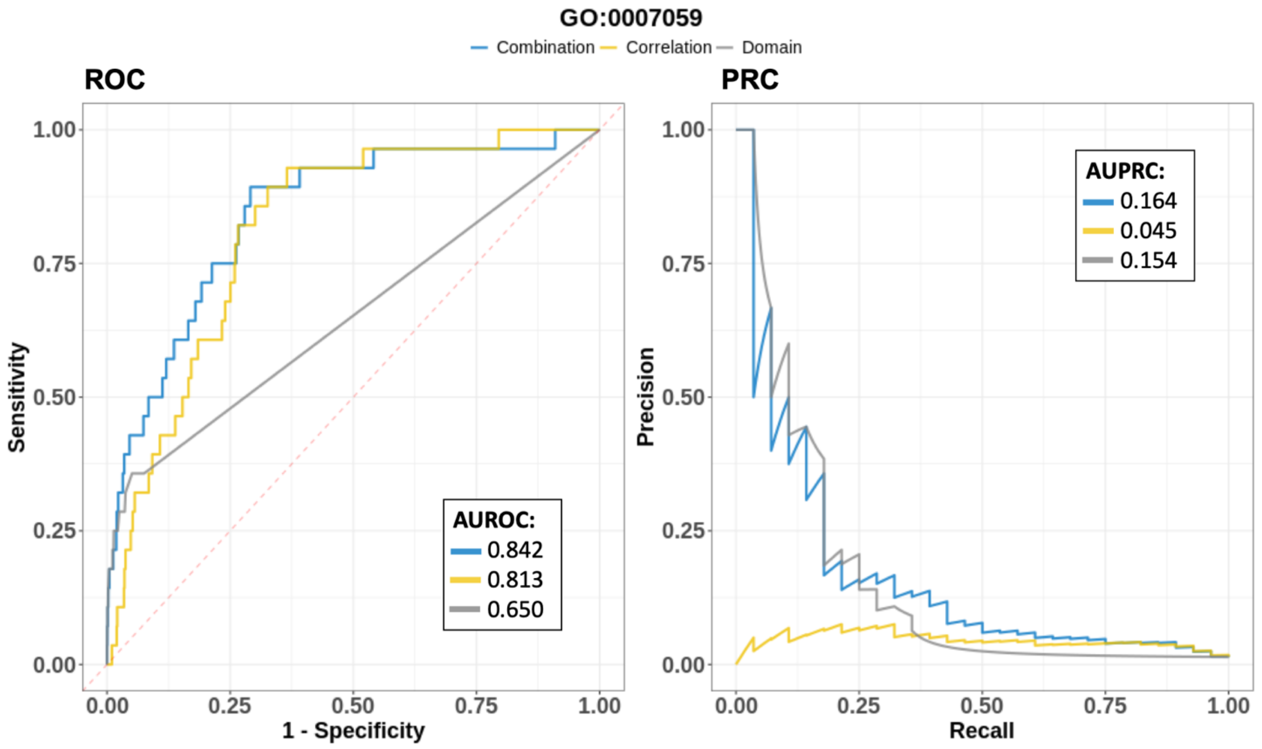

Figure S7: ROC (left) and PRC (right) curves when predicting the genes annotated to the function GO:0007059("chromosome segregation") by applying the three methods: Combination (blue), Correlation (yellow) and Domains (grey). The Correlation method (yellow) is more sensitive (AUROC = 0.813 and AUPRC = 0.045) compared with the precise Domain-based regression method (grey, AUROC = 0.650 and AUPRC = 0.154). The Combination method (blue) outperforms both the Correlation and the Domain-based regression method in the two aspects (AUROC = 0.842 and AUPRC = 0.164).

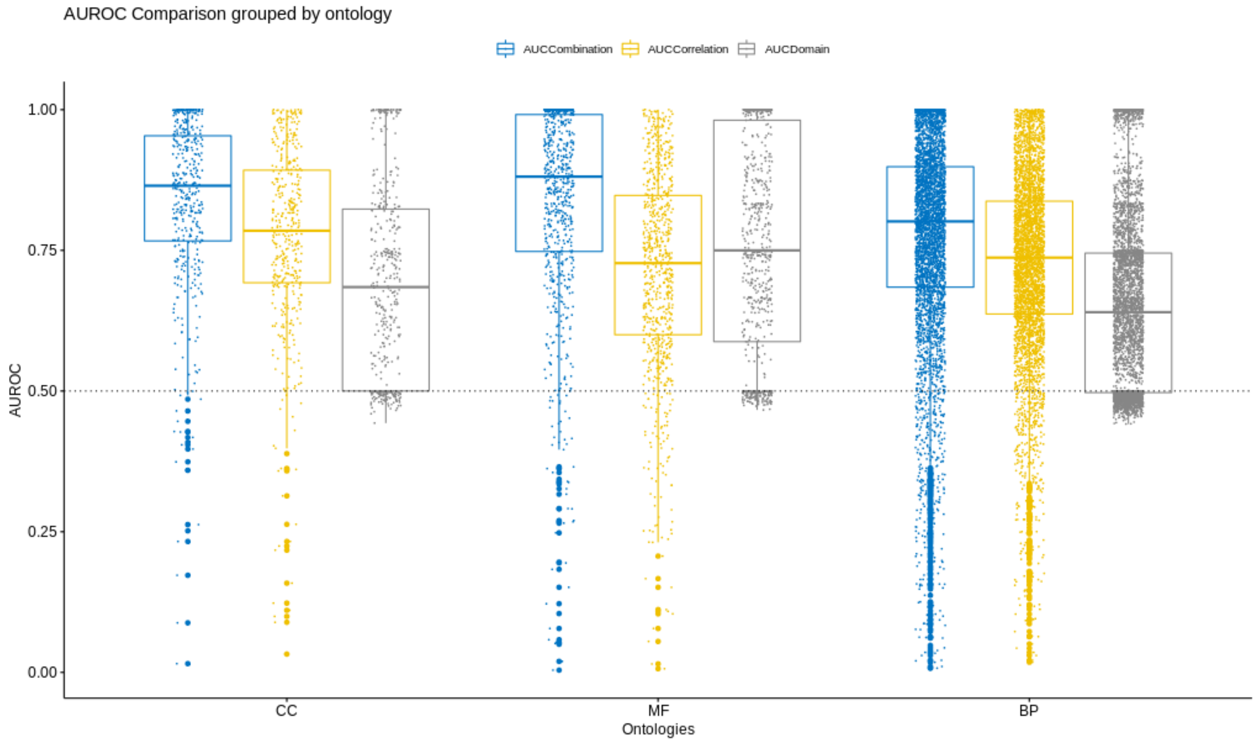

Figure S8: AUROCs comparison, depending on the ontology of each GO term. Blue boxplot corresponds to Combination method, yellow to Correlation method and grey to Domain-based regression. A dotted black line is included to show the baseline for a random classifier (AUROC = 0.5).

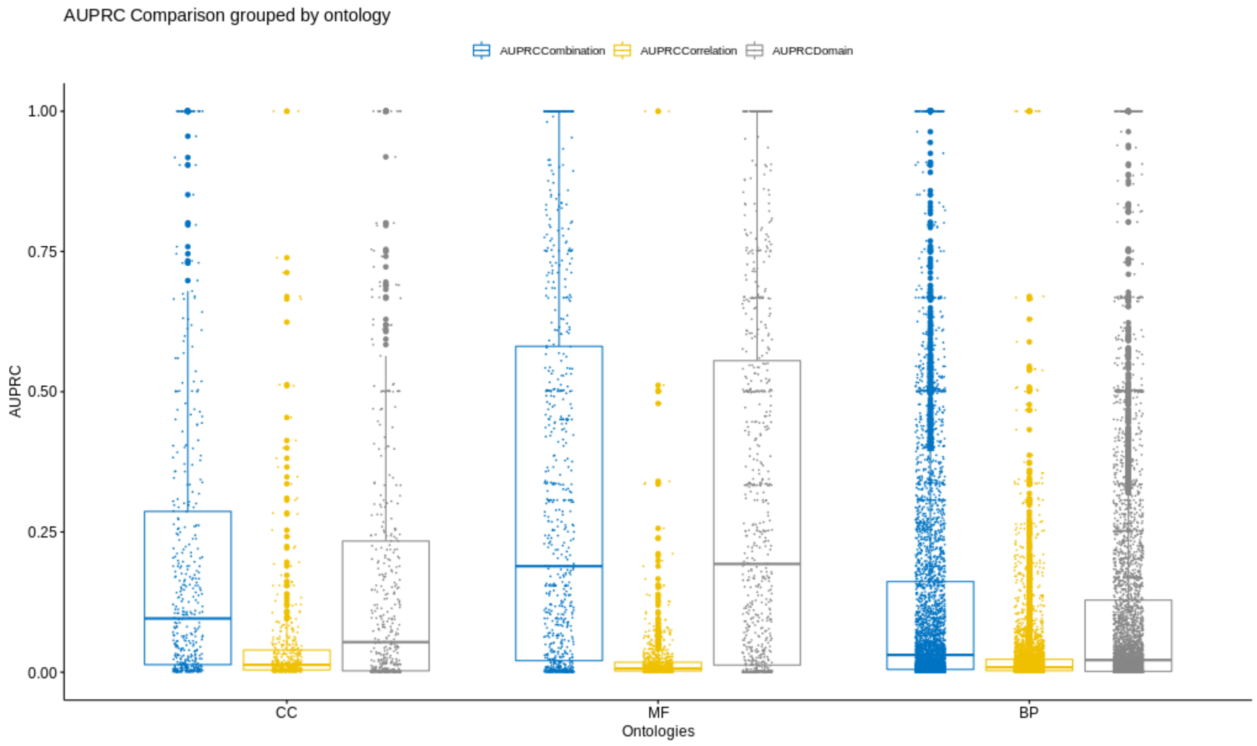

Figure S9: AUPRC comparison, depending on the ontology of each GO term. Legend as for SF 5 (blue boxplots are combination method, yellow are Correlation method and grey are Domain-based regression).

Table S1. *Pfam and InterPro description of the protein domains of unknown function (DUF) used in ISOGO (first 4 columns) and the description of the top 5 GO terms with the most reliable predictions in terms of AUROC for which the Pfam is been used as a predictor variable (last 3 columns).*

Pfam	DUF	Interpro	Interpro description	GO *term*	GO description	AUROC
PF06327	DUF1053	IPR001054	Adenylyl cyclase class-3/4/guanylyl cyclase	GO:0004016	adenylate cyclase activity	0.9997
		IPR009398	Adenylate cyclase, conserved domain	GO:0031683	G-protein beta/gamma-subunit complex binding	0.9965
		IPR018297	Adenylyl cyclase class-4/guanylyl cyclase, conserved site	GO:0030145	manganese ion binding	0.8659*
		IPR029787	Nucleotide cyclase	GO:0005902	microvillus	0.8367*
				GO:0016849	phosphorus-oxygen lyase activity	0.8332*
PF08953	DUF1899	IPR001680	WD40 repeat	GO:0048521	negative regulation of behavior	1.0000
		IPR015048	Domain of unknown function DUF1899	GO:0050922	negative regulation of chemotaxis	1.0000
		IPR015049	Trimerisation motif	GO:0035767	endothelial cell chemotaxis	0.9995
		IPR017986	WD40-repeat-containing domain	GO:1900027	regulation of ruffle assembly	0.9995
		IPR019775	WD40 repeat, conserved site	GO:0048365	Rac GTPase binding	0.9987
PF11878	DUF3398	IPR001849	Pleckstrin homology domain	GO:0005089	Rho guanyl-nucleotide exchange factor activity	1.0000
		IPR010703	Dedicator of cytokinesis, C-terminal	GO:0048365	Rac GTPase binding	0.9987
		IPR011993	PH domain-like	GO:0090630	activation of GTPase activity	0.9878
		IPR016024	Armadillo-type fold	GO:0017048	Rho GTPase binding	0.9215*
		IPR021816	Dedicator of cytokinesis C/D, N-terminal	GO:0045165	cell fate commitment	0.8873*
		IPR027007	DHR-1 domain			
		IPR027357	DHR-2 domain			
PF11938	DUF3456	IPR021852	Domain of unknown function DUF3456	GO:0034663	endoplasmic reticulum chaperone complex	0.9950
		IPR008139	Saposin B type domain	GO:2001273	regulation of glucose import in response to insulin stimulus	0.9841
		IPR000742	EGF-like domain	GO:1900076	regulation of cellular response to insulin stimulus	0.8661*
		IPR002049	Laminin EGF domain	GO:0002377	immunoglobulin production	0.8033*
		IPR009030	Growth factor receptor cysteine-rich domain	GO:0003279	cardiac septum development	0.7700*
		IPR013032	EGF-like, conserved site			
		IPR000152	EGF-type aspartate/asparagine hydroxylation site			
		IPR001881	EGF-like calcium-binding domain			
		IPR006212	Furin-like repeat			
		IPR018097	EGF-like calcium-binding, conserved site			
PF12130	DUF3585	IPR001715	Calponin homology domain	GO:0097320	plasma membrane tubulation	1.0000
		IPR001781	Zinc finger, LIM-type	GO:0055038	recycling endosome membrane	0.9962
		IPR002938	FAD-binding domain	GO:0030042	actin filament depolymerization	0.9921
		IPR022735	Domain of unknown function DUF3585	GO:0042805	actinin binding	0.9905
		IPR023753	FAD/NAD(P)-binding domain	GO:0005923	bicellular tight junction	0.9377*
		IPR019448	EEIG1/EHBP1 N-terminal domain			

		IPR016103	ProQ/FinO domain			
PF12417	DUF3669	IPR001909	Krueppel-associated box	GO:0001227	transcriptional repressor activity, RNA polymerase II transcription regulatory region sequence-specific DNA binding	0.8910*
		IPR007087	Zinc finger, C2H2	GO:0001078	transcriptional repressor activity, RNA polymerase II proximal promoter sequence-specific DNA binding	0.8579*
		IPR015880	Zinc finger, C2H2-like	GO:0031625	ubiquitin protein ligase binding	0.7563*
		IPR022137	Protein of unknown function DUF3669, zinc finger protein	GO:0044389	ubiquitin-like protein ligase binding	0.7157*
				GO:1901214	regulation of neuron death	0.6289*
PF12473	DUF3694	IPR000253	Forkhead-associated (FHA) domain	GO:0007274	neuromuscular synaptic transmission	0.9995
		IPR001752	Kinesin motor domain	GO:0047496	vesicle transport along microtubule	0.9847
		IPR001849	Pleckstrin homology domain	GO:0030695	GTPase regulator activity	0.9088*
		IPR008984	SMAD/FHA domain	GO:0005875	microtubule associated complex	0.8776*
		IPR011993	PH domain-like	GO:0072384	organelle transport along microtubule	0.8511*
		IPR019821	Kinesin motor domain, conserved site			
		IPR022140	Kinesin-like KIF1-type			
		IPR022164	Kinesin-like			
		IPR027417	P-loop containing nucleoside triphosphate hydrolase			
		IPR006020	PTB/PI domain			
		IPR000195	Rab-GTPase-TBC domain			
		IPR000938	CAP Gly-rich domain			
PF12480	DUF3715	-	-	-	-	-
PF15371	DUF4599	-	-	-	-	-

** GO terms with an AUROC less than 0.95*

GO PREDICTIONS FOR GENES WITH KNOWN ISOFORM SPECIFIC FUNCTIONS.

Table S2. *GO terms shown in figures 4A, 4C, and 4E.*

Origin	*GO term*	*GO description*
Figure 4A	*GO:0008625*	*Extrinsic apoptotic signaling pathway via death domain receptors*
	GO:0008630	*Intrinsic apoptotic signaling pathway in response to DNA damage*
	GO:0097191	*Extrinsic apoptotic signaling pathway*
	GO:0097193	*Intrinsic apoptotic signaling pathway*
	GO:1902041	*Regulation of extrinsic apoptotic signaling pathway via death domain receptors*
	GO:1902042	*Negative regulation of extrinsic apoptotic signaling pathway via death domain receptors*
	GO:2001234	*Negative regulation of apoptotic signaling pathway*
	GO:2001236	*Regulation of extrinsic apoptotic signaling pathway*
	GO:2001237	*Negative regulation of extrinsic apoptotic signaling pathway*
	GO:1902043	*Positive regulation of extrinsic apoptotic signaling pathway via death domain receptors*
Figure 4C	*GO:0008625*	Extrinsic apoptotic signaling pathway via death domain receptors
	GO:0097191	*Extrinsic apoptotic signaling pathway*
	GO:0097194	*Execution phase of apoptosis*
	GO:1902041	*Regulation of extrinsic apoptotic signaling pathway via death domain receptors*
	GO:2001236	*Regulation of extrinsic apoptotic signaling pathway*
Figure 4E	*GO:0017156*	*Calcium ion regulated exocytosis*
	GO:0017157	*Regulation of exocytosis*
	GO:0045055	*Regulated exocytosis*
	GO:0045921	*Positive regulation of exocytosis*
	GO:0000149	*SNARE binding*
	GO:0031201	*SNARE complex*
	GO:0035493	*SNARE complex assembly*

INDIRECT TRANSCRIPTOME-WIDE VALIDATION: APPRIS AND CAFA3

APPRIS:

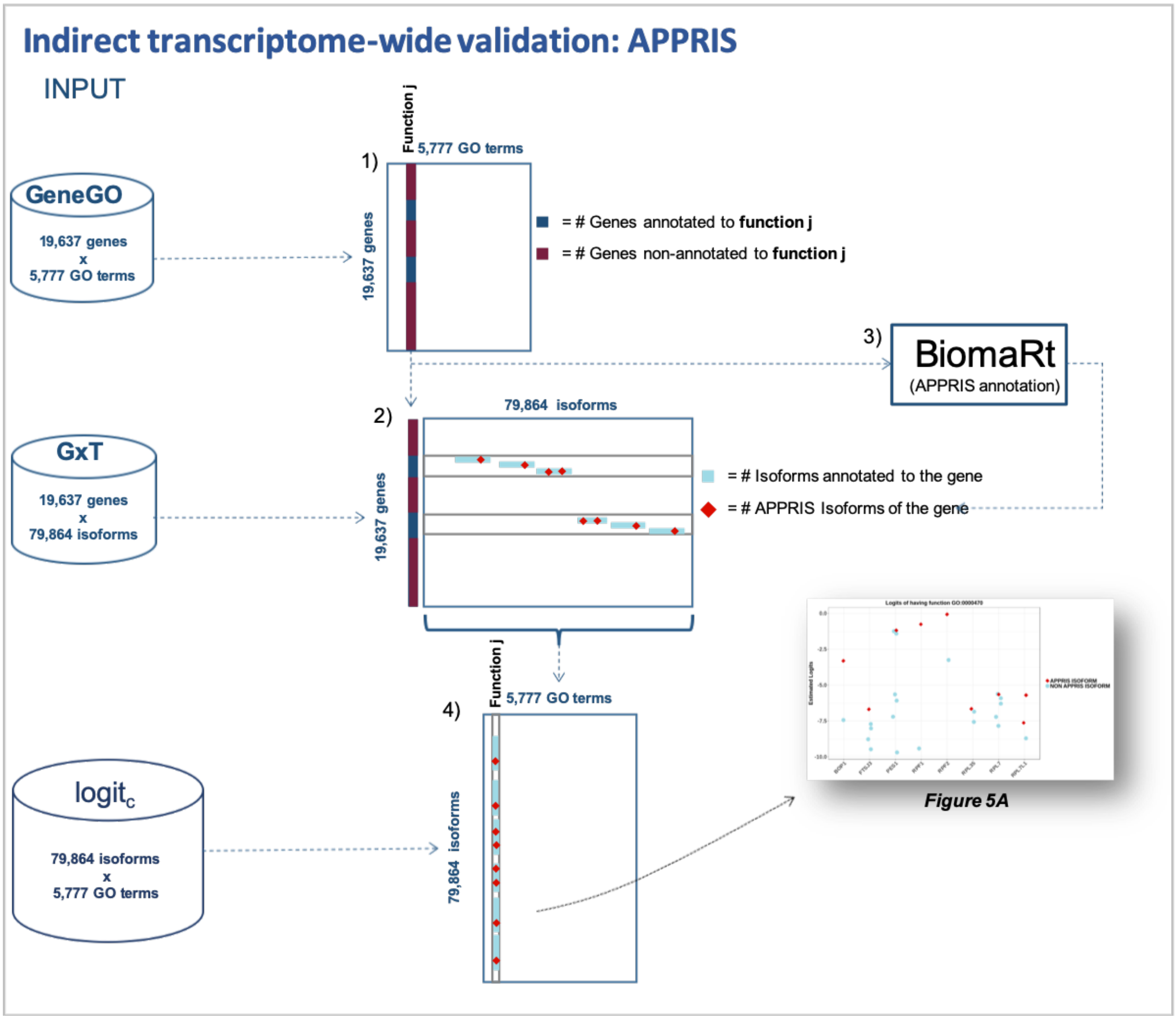

Figure S10: APPRIS [2] indirect transcriptome-wide validation. For each GO term: ***1)****: we selected its annotated genes. In 1) annotated genes are shaded in blue and non-annotated in red ;* ***2)****: we selected for these genes its corresponding isoforms;* ***3)****:by using BiomaRt R package [3] we distinguished the APPRIS isoforms and the non-APPRIS isoforms. In 2) the splice variants that belong to the selected genes are marked in blue and the major isoforms are distinguished with red diamonds;* ***4)****: we tested, among all the isoforms of the selected genes, whether the APPRIS isoforms were the most likely to perform the function.*

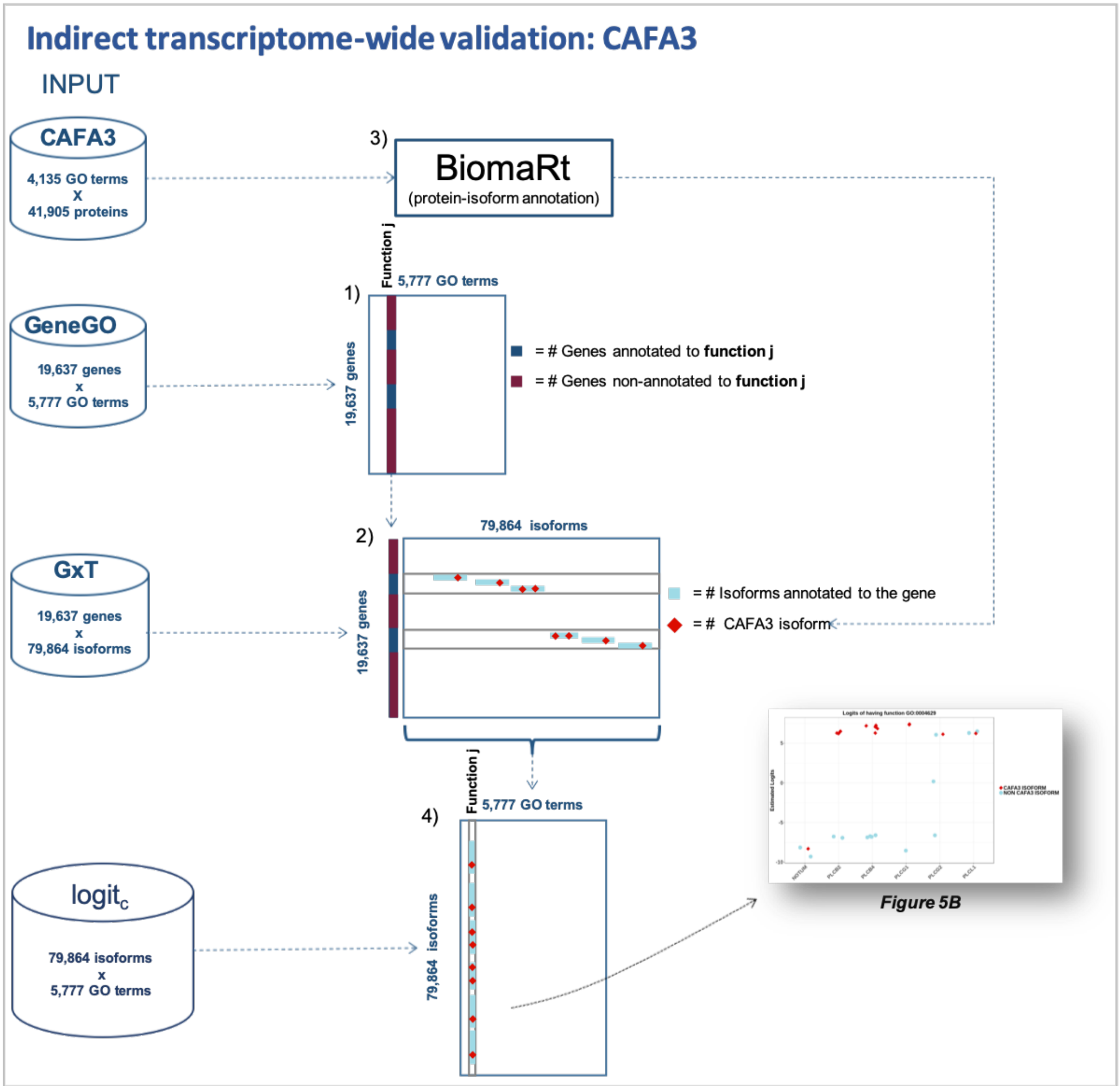

Figure S11: CAFA3 indirect transcriptome-wide validation. For each GO term: ***1)****: we selected its annotated genes. In 1) annotated genes are shaded in blue and non-annotated in red;* ***2)****: we selected for these genes its corresponding isoforms;* ***3)****:by using BiomaRt R package* [3] *we related the protein included in the CAFA3 challenge with isoforms, achieving a dataset were protein, function and isoform are merged i.e. specific function are related with specific isoforms;* ***4)****: we tested, among all the isoforms of the genes, whether the 'CAFA3' isoforms were the most likely to perform the function.*

DISCUSSION

Table S3. *Overall performance of each method using data from CCLE. AUROC column shows the median of the AUROC; AUPRC displays the median of the AUPRC for each method; #terms column indicates the number of total functions with perfect performance. Domain-based regression displays the same results shown in Table 1 as it does not depend on the expression data. We included Domain-based regression for sake of completeness.*

Method	*AUROC*	*AUPRC*	*#terms*
Correlation method	*0.706*	*0.0072*	*4*
Domain-based regression	*0.657*	*0.0293*	*147*
Combination method	*0.791*	*0.0385*	*186*

Table S4. *Overall performance of each method using data from Normal tissues. AUROC column shows the median of the AUROC; AUPRC displays the median of the AUPRC for each method; #terms column indicates the number of total functions with perfect performance. Domain-based regression desplays the same results shown in Table 1 as it does not depend on the expression data.*

Method	*AUROC*	*AUPRC*	*#terms*
Correlation method	*0.725*	*0.0082*	*5*
Domain-based regression	*0.657*	*0.0293*	*147*
Combination method	*0.811*	*0.0409*	*198*

Coherence technique

In some predictions, the probability assigned to an ancestor is not larger than the ones assigned to any of its descendants. Several works tried to explicitly consider the relationship between functional classes [4][5][6]. These works propose different methods or heuristics to ensure the coherence [1][7][8][9] of the ontology, so that, the predicted probability of any given *GO term* is equal or smaller than that of its parents.

Our method to ensure coherence uses the variance of the estimate to decide how to update the logits. If variance is low, then the algorithm is *confident* of the estimated likelihood. Conversely, if variance is high, then the predicted estimate is not as reliable. This intuition can be expressed mathematically in terms of maximizing likelihood. As logistic regression logits can be considered to follow –asymptotically– a normal distribution, the proposed minimization problem is:

$$\min_{a_i} \sum_{i=1}^{n} \frac{(a_i - b_i)^2}{\sigma_i^2}$$

Subject to $a_i \geq a_j$ for all a_j that are children of a_i in GO hierarchy.

In this formula, *n* is the number of *GO terms*. **a** is a *nx1* vector, whose entries are the logits after coherence (i.e. the unknown variables of the optimization problem). **b** is a *nx1* vector whose entries are the estimated logits before applying coherence (i.e. the output of the combination method). **σ** is a *nx1* vector with the standard deviation of the **b** estimates. The restriction ensures that the logits of the parents must be larger or equal to the logits of its children.

The system of equations contains 1,428 restrictions and 748 functions, for Cellular Component; 13,633 restrictions and 6,074 functions, for Biological Process and 1,474 restrictions and 1,107 functions for Molecular Function. In turn, these three optimizations must be solved for each gene or isoform. This is a convex quadratic programming problem that can be solved by using *Rcplex* [10], an R package that interface to *Cplex* [11].

Isoforms of the same gene with opposite functions

ITSN1 is a gene with the annotations to two opposite functions, namely, GO: 0043065 ("*positive regulation of apoptosis*") and GO:0043066 ("*negative regulation of apoptosis*"). ITSN1 short isoform has shown to be a negative regulator of apoptosis whereas its long isoform has the opposite function [12,13]. ISOGO predicted this phenomenon assigning an increased likelihood of positive regulation of apoptosis to the long isoform. Similarly, an increased likelihood of negative regulation of apoptosis was assigned to the short isoform (Figure S12).

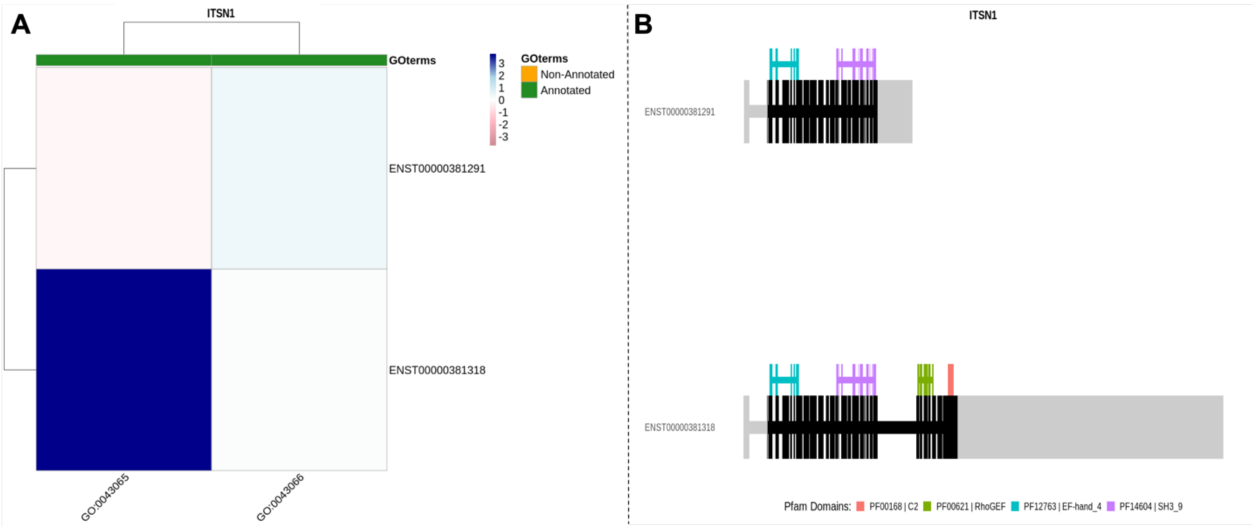

Figure S12: Panel A) shows heatmaps of the difference between the ISOGO and the expected logits of an isoform having a function, where larger values are represented in blue and smaller values in red. Panel B) shows the isoform structure and position of protein domains for the short and long isoform of ITSN1

1. Li W, Kang S, Liu C-C, et al. High-resolution functional annotation of human transcriptome: predicting isoform functions by a novel multiple instance-based label propagation method. Nucleic Acids Res. 2014; 42:e39–e39

2. Rodriguez JM, Maietta P, Ezkurdia I, et al. APPRIS: annotation of principal and alternative splice isoforms. Nucleic Acids Res. 2013; 41:D110–D117

3. Durinck S, Spellman PT, Birney E, et al. Mapping identifiers for the integration of genomic datasets with the R/Bioconductor package biomaRt. Nat. Protoc. 2009; 4:1184–91

4. Eisner A, Feierl G, Gorkiewicz G, et al. High Prevalence of VanA-Type Vancomycin-Resistant Enterococci in Austrian Poultry. Appl. Environ. Microbiol. 2005; 71:6407–6409

5. Shahbaba B, Neal RM. Gene function classification using Bayesian models with hierarchy-based priors. BMC Bioinformatics 2006; 7:448

6. Gruber J, Manninga H, Tuschl T, et al. Specific RNAi mediated gene knockdown in zebrafish cell lines. RNA Biol. 2006; 2:101–5

7. Panwar B, Menon R, Eksi R, et al. Genome-wide functional annotation of human protein-coding splice variants using multiple instance learning. J. Proteome Res. 2016; 15:1747–1753

8. Notaro M, Schubach M, Robinson PN, et al. Prediction of Human Phenotype Ontology terms by means of hierarchical ensemble methods. BMC Bioinformatics 2017; 18:449

9. Jiang Y, Oron TR, Clark WT, et al. An expanded evaluation of protein function prediction methods shows an improvement in accuracy. Genome Biol. 2016;

10. Bravo HC, Hornik K, Theussl S. Rcplex: R Interface to CPLEX.

11. Ibm. IBM ILOG CPLEX Optimization Studio CPLEX User's Manual. 2011;

12. Predescu SA, Predescu DN, Knezevic I, et al. Intersectin-1s regulates the mitochondrial apoptotic pathway in endothelial cells. J. Biol. Chem. 2007; 282:17166–17178

13. Shao Y, Chong W, Liu X, et al. Alternative splicing-derived intersectin1-L and intersectin1-S exert opposite function in glioma progression. Cell Death Dis. 2019; 10:
